# Towards uniformity in the definition of acceptable mismatches for highly sensitized patients

**DOI:** 10.1111/tan.13607

**Published:** 2019-06-19

**Authors:** Mian Chen, Yvonne Zoet, Dave Roelen, Jaume Martorell, Derek Middleton, Antonij Slavcev, Aliki Iniotaki, Frans Claas, Susan Fuggle

**Affiliations:** ^1^ Oxford Transplant Centre, Nuffield Department of Surgical Sciences Oxford University Hospitals, Churchill Hospital Oxford UK; ^2^ Eurotransplant Reference Laboratory, Department of Immunohematology and Blood Transfusion Leiden University Medical Center Leiden the Netherlands; ^3^ Department of Immunohematology and Blood transfusion Leiden University Medical Center Leiden the Netherlands; ^4^ Hospital Clínic Barcelona Barcelona Spain; ^5^ Transplant Immunology Laboratory Royal Liverpool and Broadgreen University Hospital Liverpool UK; ^6^ Institute of Infection and Global Health University of Liverpool Liverpool UK; ^7^ Department of Immunogenetics Institute for Clinical and Experimental Medicine Prague Czech Republic; ^8^ National Tissue Typing Center General Hospital of Athens “G. Gennimatas” Athens Greece; ^9^ Organ Donation and Transplantation NHS Blood and Transplant Bristol UK

**Keywords:** acceptable mismatch, highly sensitized, HLA antibody, kidney transplant, Luminex, multicentre studies

## Abstract

The Eurotransplant (The Eurotransplant International Foundation) acceptable mismatch programme has been shown to be a successful tool to enhance transplantation of highly sensitized patients(HSPs). However, patients with rare HLA phenotypes in relation to the Eurotransplant donor population remain on the waiting list. EUROSTAM is an European Union funded project to explore the feasibility of a Europe‐wide acceptable mismatch programme enabling transplantation of HSPs with rare HLA phenotypes within their own organ exchange organization. The present study, which forms part of the EUROSTAM project, assesses the differences in the practices of the laboratories in different countries with respect to their HLA antibody profiling and risk adverseness. In the serum exchange exercises of 18 samples, a high level of variability has been shown in both assays and interpretation of results. In the data exchange exercise when all participants were given the same Luminex raw data for analysis, a high degree of consensus was reached where the median fluorescent intensity values of beads were <500 and >2000 for standard single antigen bead assays, or <500 and >5000 for assignment of acceptable mismatches. The risk adverseness analysis has showed distinct patterns of attitudes towards the perceived risks based on HLA antibody assay results, most probably influenced by the local protocols of the clinical transplant programme of each laboratory. In order to ensure fairness and maintain consistencies of organ exchange among partner transplant centres, a centralized facility will be instrumental for a uniform definition of acceptable mismatches.

**Abbreviations**
AM: acceptable mismatch
EC: The European Commission
EU: The European Union
Eurotransplant: The Eurotransplant International Foundation
HSP: highly sensitized patient
LUMC: Leiden University Medical Center
MFI: median fluorescent intensity
SAB: single antigen beads
UM: unacceptable mismatch

## 1 INTRODUCTION

The presence of donor‐specific HLA antibodies, especially when detectable in complement‐dependent cytotoxicity tests, is considered a contra‐indication for kidney transplantation.^1^, ^2^ Exclusion of donors with HLA antigens towards which a patient has formed antibodies improves outcome. However, such policy will lead to accumulation of highly sensitized patients (HSPs) on the waiting list. These patients have broadly reactive HLA antibodies due to immunization by previous pregnancies, blood transfusions or transplants. Special strategies are required to enhance transplantation of (HSPs). A very successful strategy is the acceptable mismatch programme of Eurotransplant (The Eurotransplant International Foundation), which positively identifies those HLA antigens towards which the patient has not made any clinical relevant antibodies.^3^, ^4^, ^5^, ^6^ Donor selection for these patients is based on compatibility with the combination of the patient's own HLA antigens and the acceptable mismatches. Once such a donor becomes available within Eurotransplant a donor kidney is mandatorily shipped to the HSP. This policy has enhanced transplantation of HSPs with excellent long‐term outcome. However, this strategy does not work for patients with very rare HLA types in relation to their donor population. For these patients no compatible donor can be found within the Eurotransplant donor pool. As the HLA phenotypes differ among the different European populations,^7^, ^8^ we wondered whether extension of the potential donor pool with donors derived from other European organ exchange organizations would be beneficial for transplantation of these patients. EUROSTAM is a project started in late 2012 (http://eurostam.eu) and funded by the European Commission (FP7‐HEALTH Collaborative project). The major objective of the partners in this project is to analyse the feasibility and requirements for a Europe‐wide acceptable mismatch programme to enhance transplantation of patients with rare HLA phenotypes in their own population. One of the main aspects is to evaluate the feasibility and possible logistics of managing and sharing the antibody profile of HSPs in order to define the acceptable mismatches. This study was designed to assess the differences in the practices of HLA antibody definition and risk stratification for transplant in the six partners of this project.

## 2 MATERIALS AND METHODS

### 2.1 Reagents

HLA antibody definitions were performed using either the LABScreen class I (LS1A04) and class II (LS2A01) single antigen bead (SAB) kits from One Lambda (Canoga Park, California) or Lifecodes class I (LSA1) and class II (LSA2) SAB kits from Immucor Transplant Diagnostics (Stamford, Connecticut). Each participant laboratory follows its own routine Luminex assay protocols.

### 2.2 Serum sample exchange

Serum samples selected by the Eurotransplant reference laboratory at the Leiden University Medical Center (LUMC) were sent to the partner laboratories (Athens, Barcelona, Leiden, Liverpool, Oxford and Prague) for testing and analysis of specificities and designation of acceptable mismatches according to local standard operating procedures and relevant local policies. Six samples were sent each year in 2013, 2014 and 2015. The 2015 exchange was based on the experience from the 2013 and 2014 pilot studies. The 2015 samples were tested and analysed in 5/6 laboratories (Laboratories A‐E) by LABScreen SAB. Lifecodes class I and II SAB (LSA1 and LSA2) was also performed in 3/6 laboratories (Laboratories A, B, F). For each serum sample, each participating laboratory assigned all HLA specificities covered by the kits as either “positive” or “negative” in each of the assays. Based on the results of the testing, each HLA specificity was also designated as an “acceptable mismatch” (AM) or an “unacceptable mismatch” (UM) according to local criteria. The results were collated centrally and analysed for concordance.

### 2.3 Data exchange exercise

**Figure 1:**
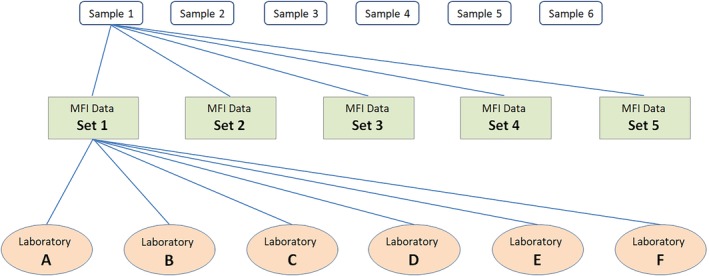
Data exchange exercise. Samples 1 to 6 were tested by five laboratories in the 2015 sera exchange exercise. Five sets of MFI data were generated for each sample (sets 1‐5). The data were sent to all of the six participating laboratories (A‐F) for analysis. Each laboratory assigned the HLA‐A, ‐B, ‐C, ‐DR and ‐DQ antibody specificities and designated AM for every set of results for every sample

There was also an exercise whereby data were exchanged. Median fluorescence intensity (MFI) values from the six samples tested by the five laboratories using the LABScreen SAB assay in the 2015 serum exchange generated five sets of data (sets 1‐5). The data were anonymized and sent to all of the six participating laboratories. Each laboratory assigned the HLA‐A, ‐B, ‐C, ‐DR and ‐DQ antibody specificities and AM based on each set of data for each sample (Figure 1). Results of the analyses were submitted centrally, collated and analysed.

#### 2.3.1 Analysis

**Figure 2:**
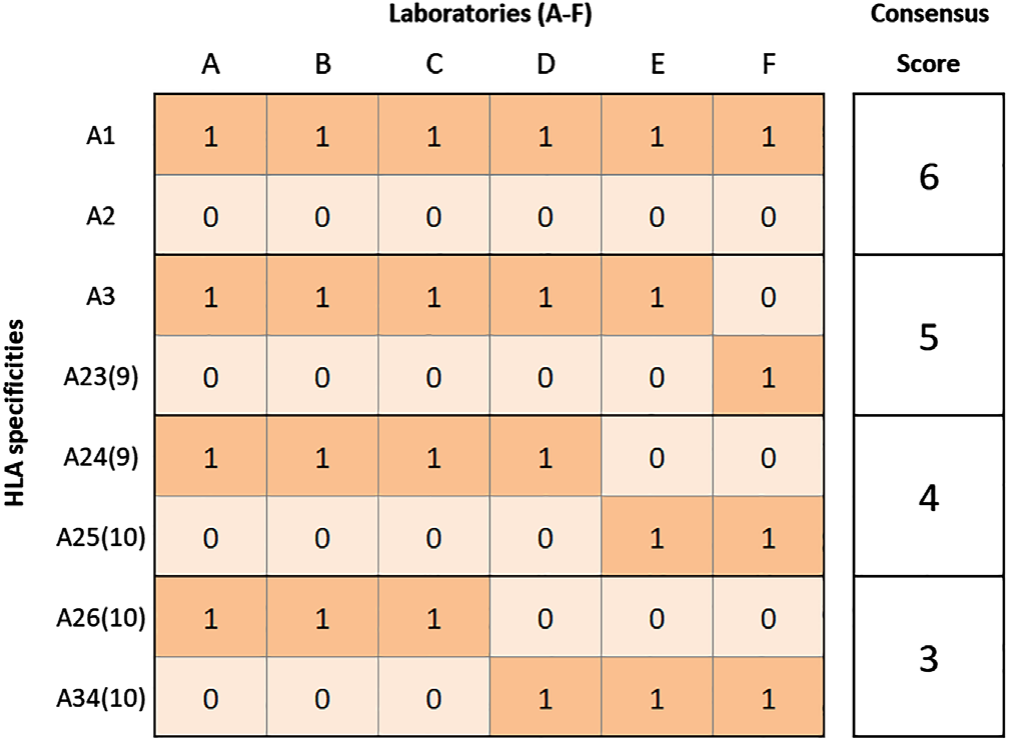
Illustration of the consensus score calculation. In the data exchange exercises, when analysing a sample the laboratories assigned antibody specificities as either positive or negative, which were then coded as 1 or 0, respectively. Columns labelled A‐F give the examples of results from six participating laboratories (A‐F). The highest consensus score is 6, when all laboratories agree. The lowest consensus score is 3, where only half of the laboratories are in agreement. For example in the illustration above, HLA‐A1 and HLA‐A2 both have a consensus score of 6. HLA‐A1 was assigned as positive and HLA‐A2 was assigned as negative by all laboratories. The same consensus score calculation was applied to the designation of acceptable mismatches

First, the consensus scores in the interpretation of specificities from one set of MFI results were calculated. Consensus scores range from 3‐6, where 6 is full consensus and 3 is no consensus. Figure 2 shows how the consensus scores were calculated for each specificity in a sample. This calculation was also performed for the assignment of acceptable mismatches.

In order to explore how MFI values influenced the decision to assign an antibody specificity or designate an AM, further analysis was performed. In this analysis, each specificity was mapped to a baseline MFI value from one set of data. When an HLA specificity was represented by more than one allele, the value from the bead with the highest MFI value was used. For example, in LABScreen SAB class I kit (LS1A04), HLA‐A2 is represented by beads coated with HLA‐A*02:01, A*02:03 and A*02:06 and the highest MFI value of these three beads was given to HLA‐A2 in the analysis. Another example is in the LABScreen SAB class II kit (LS2A01), where HLA‐DQB1*03:01 is represented by five beads with different HLA‐DQA1 alleles, the highest MFI value of these five beads was given to DQ7 for analysis in this study.

An assumption was made that antibody specificities directed against HLA‐DQ were against the HLA‐DQB chain. Inclusion of HLA‐DQA was beyond the scope of the analysis.

#### 2.3.2 Data analysis and visualisation

For the initial data collection and conversion Microsoft Excel and Visual Basic for Application (VBA) (Microsoft, Redmond, Washington) were used. All of the subsequent data processing, analysis and visualisation were performed by bespoke algorithms using the Python programming language (Python Software Foundation, Wilmington, Delaware) and third party libraries, including Pandas^9^ and Matplotlib.^10^

## 3 RESULTS

### 3.1 Serum sample exchange analyses

Following the pilot exchanges in 2013 and 2014, six further samples were tested in 2015. The results from SAB testing were received from all laboratories, collated and analysed for concordance. Concordance was defined when the presence or absence of a specificity was reported by all of the laboratories performing the assay. The level of concordance between laboratories in defining specificities at HLA‐A, ‐B, ‐C, ‐DR and ‐DQB is shown in Table 1.

**Table 1:** Results from 2015 sera exchange

Concordance in assignment of specificities							
	HLA‐A (n = 21) (%)	HLA‐B (n = 46) (%)	HLA‐C (n = 15) (%)	Class I (n = 82) (%)	HLA‐DR (n = 16) (%)	HLA‐DQ (n = 7) (%)	Class II (n = 23) (%)
SAB assays^a^	52	38	38	41	50	52	51
SAB LabScreen	88	90	91	90	90	88	89
SAB Lifecodes	90	79	92	84	84	95	88

Concordance in designation of acceptable mismatches							
AM	67	68	70	68	74	60	70

a SAB results from both LabScreen and Lifecodes assay.

When the antibody specificities defined by Lifecodes and LabScreen assays were analysed together, the concordance was relatively low, ranging from 38% for HLA‐B and HLA‐C to 52% for HLA‐A and HLA‐DQ. However, when the results from each of the kits were analysed separately the level of concordance was higher. The level of concordance achieved by LabScreen ranged from 88% for HLA‐A and HLA‐DQ, to 91% for HLA‐C; and for Lifecodes from 79% for HLA‐B, to 95% for HLA‐DQ.

When the results of these assays were used to assign an acceptable mismatch the level of concordance was significantly lower, ranging from 60% for HLA‐DQ to 74% for HLA‐DR (Table 1).

### 3.2 Data exchange analyses

#### 3.2.1 Consensus score vs MFI

The serum exchange exercises showed a lack of concordance between laboratories in designating AM when based on assays performed in their own laboratory. The MFI values in Luminex assays can be influenced by factors including laboratory protocols and individual operators.^11^ Therefore, a data exchange exercise was conducted to eliminate these factors and focus on how MFI values are used in interpretation.

Five sets of MFI data for each of six samples tested using LabScreen SAB were sent to all six partner laboratories to be used to assign antibody specificities and AM. Consensus scores were calculated for each specificity on each sample as described in Section 2.

**Figure 3:**
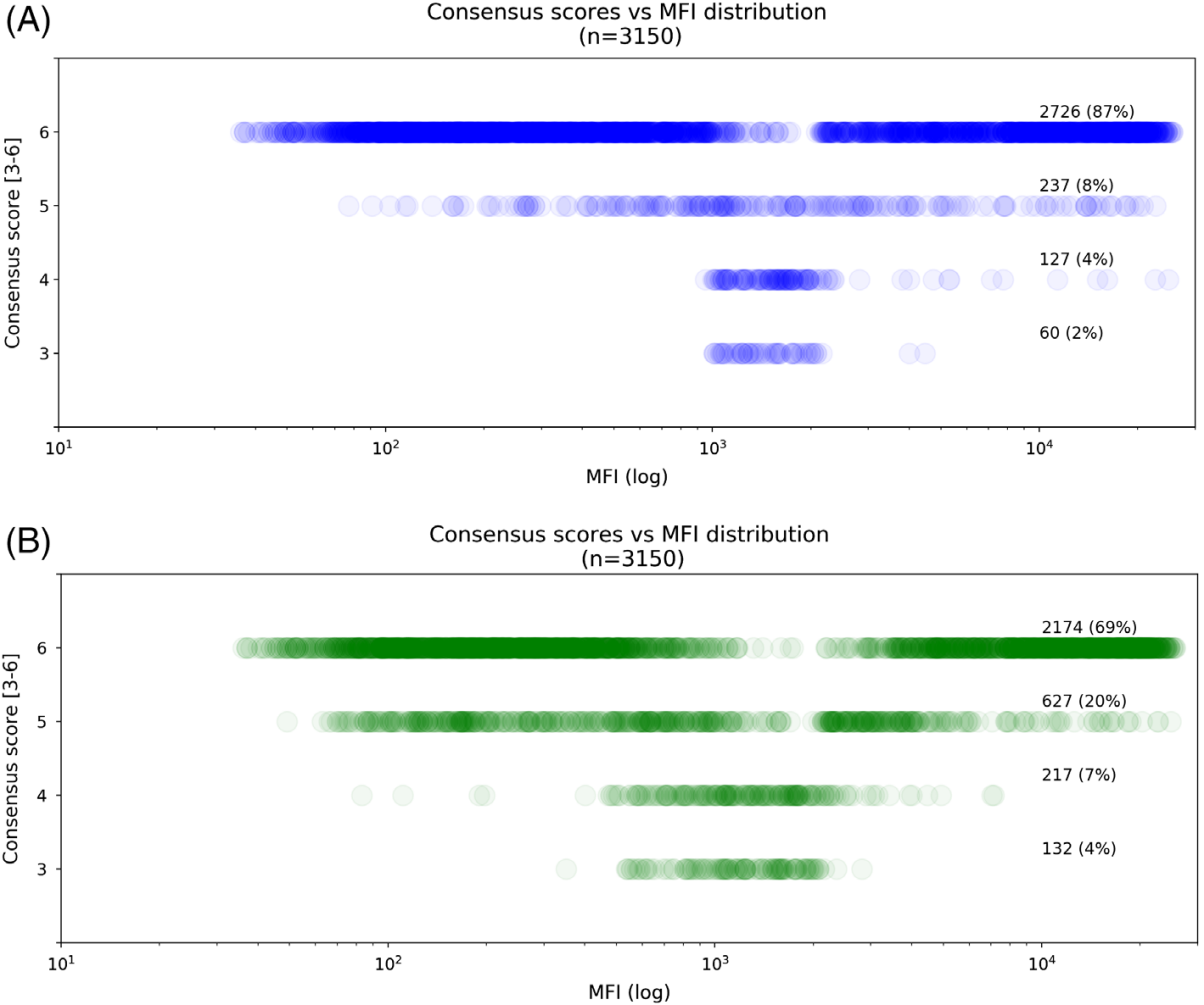
Consensus scores vs MFI distribution. This figure shows the spread of MFI values (*X* axis) associated with consensus scores 3 to 6 (*Y* axis). Each circle represents a single specificity, and higher colour density indicates a larger number of circles in that area and vice versa. A, Consensus within HLA antibody specificities assigned from LabScreen SAB assays. The figure shows a bimodal distribution of specificities having a consensus score of 6 with high levels of consensus with low and high MFI values. The majority of specificities with lower levels of consensus are distributed around MFI range 1000 to 2000. B, Consensus within acceptable mismatches designations

Exploratory data analyses were performed to understand the relationship between the consensus scores and MFI values. In Figure 3A the consensus scores of the HLA antibody specificity assignment for HLA‐A, ‐B, ‐C, ‐DR and ‐DQ were plotted against MFI values on a logarithmic scale. For the majority of specificities (2726/3150, 87%) there is complete agreement with a consensus score of 6, this applies to the MFI of <1000 or >2000. Most of the lower levels of consensus (60/3150, 2% scored 3 and 127/3150, 4% scored 4) are distributed around the MFI range 1000 to 2000.

Figure 3B shows similar trends for designation of acceptable mismatches. However, there are a lower proportion of AM where there is a consensus score of 6 (2174/3150, 69%) while the lower level consensus scores (3 and 4) are associated with a wider range of MFI values, 500 to 2500.

**Figure 4:**
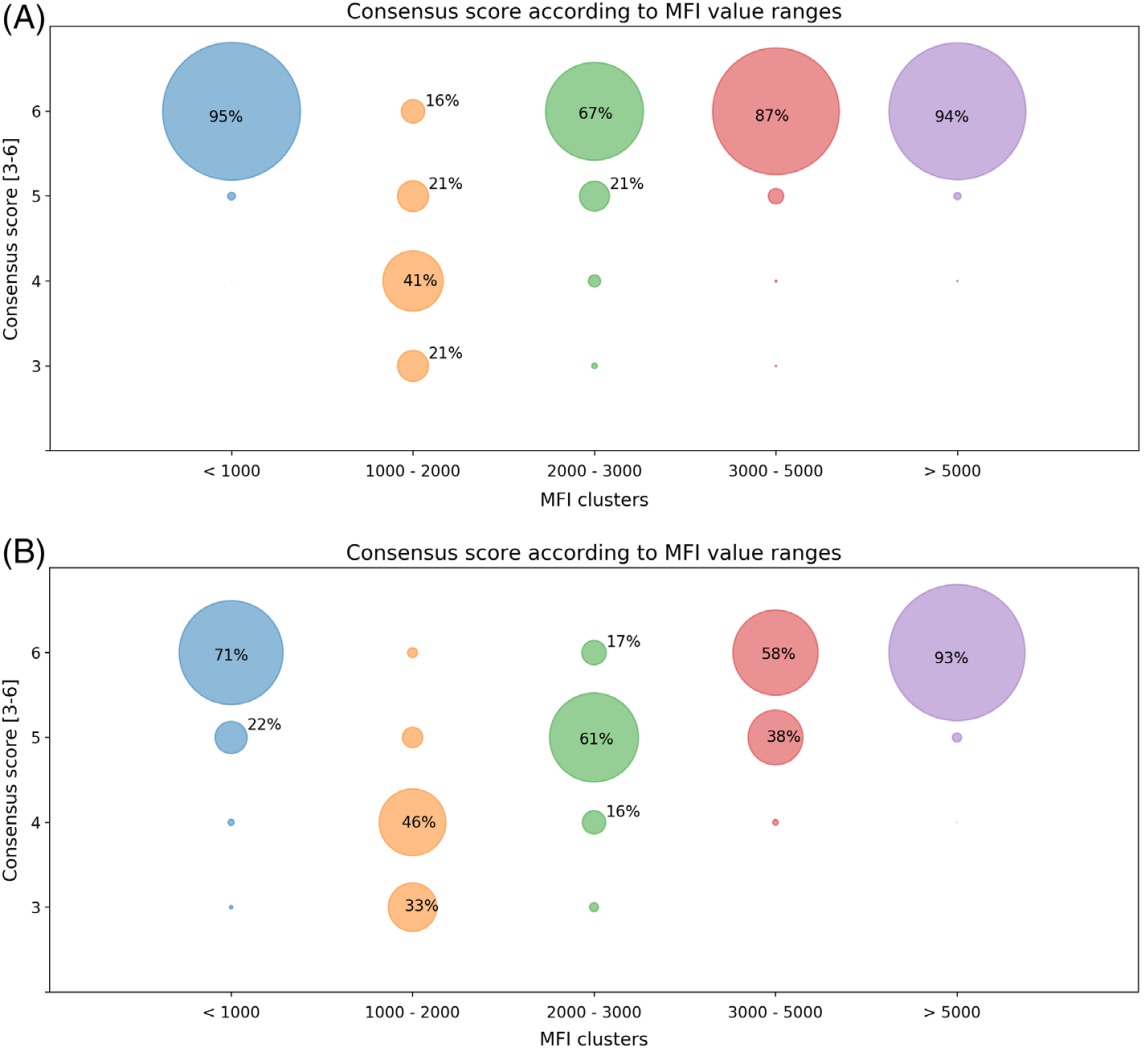
Consensus score according to MFI value ranges. Each circle represents the specificities within the MFI range (*X* axis) achieving a corresponding consensus score (*Y* axis). A, HLA antibody specificity assignment from LabScreen SAB assay. B, Acceptable mismatches designated. Most of the assignments with lower levels of consensus are in the regions with MFI values ranges from 1001 to 2000 for the SAB assay (A) and 1001 to 5000 for acceptable mismatches (B)

To further analyse the influence of MFI value on the assignment of specificities and designation of acceptable mismatches, the specificities and AM have been grouped according to MFI ranges. Figure 4A shows that in the SAB assay, when the MFI is <1000 or >5000, 95% and 94% of specificities respectively, have consensus score of 6, that is, there is full agreement. There were 587/3150 (19%) specificities within the other MFI ranges, 3001 to 5000, 2001 to 3000 and 1001 to 2000. The number of specificities reaching full consensus decreases as the MFI values decrease. For specificities within the MFI value range 1001 to 2000, only 40/249 (16%) had a consensus score of 6.

In designating AM, Figure 4B, for AM derived from MFI values >5000 there is 93% full agreement with a consensus score of 6. However, the overall level of consensus was lower than that for assigning specificities in all other MFI ranges.

There is clearly more variation between laboratories in designation of AM than in assignment of antibody specificity. In addition to a difference in the MFI cut‐offs applied, the variation may also be influenced by different clinical approaches to transplanting sensitized patients in the participating centres.

#### 3.2.2 Strategy when assigning acceptable mismatches

**Figure 5:**
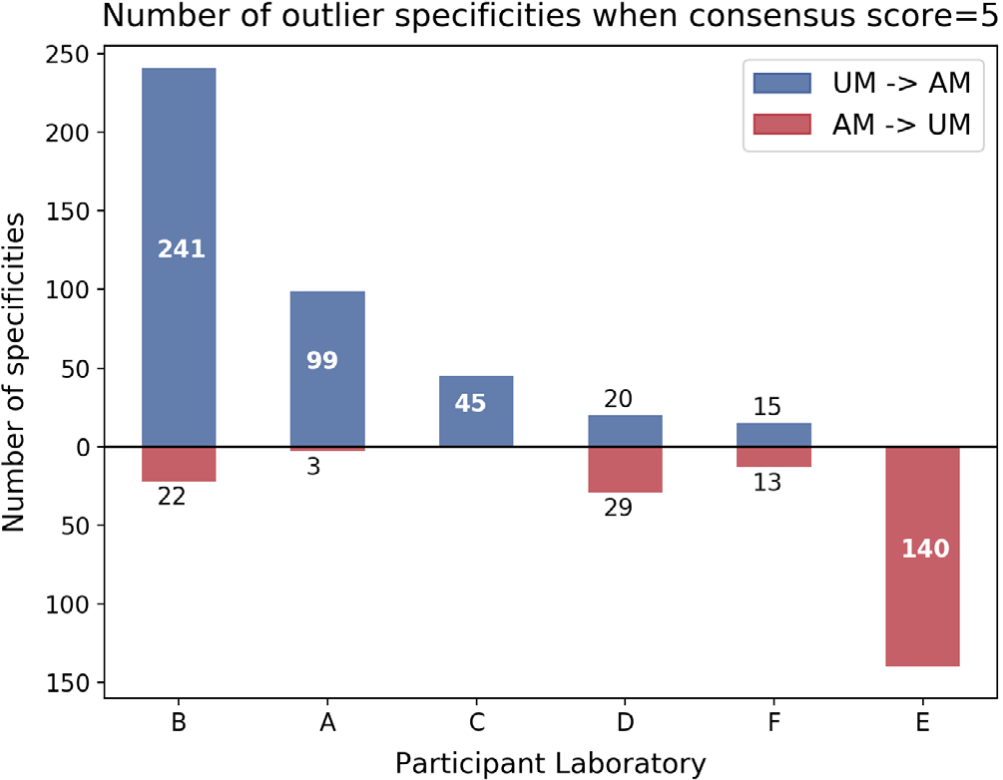
Analysis of assignment of AM/UM when there is only one outlier (consensus score = 5). Number of specificities where the consensus assignment was an UM and the outlier centre assigned an AM are shown in the top half of the figure in blue. Specificities where the consensus was an AM and the outlier centre assigned an UM are shown in red

In order to explore approaches taken to designate AM in different centres, an analysis was performed on the 607/3150 (19%) specificities where the consensus score was 5, that is, either five of six centres designated the specificity as an AM and the remaining laboratory designated an unacceptable mismatch UM or vice versa. Figure 5 shows the number of specificities differing from the consensus in each laboratory. Laboratory B designated 241 (7.7%) specificities as acceptable when the consensus was unacceptable, whereas Laboratory E designated 140 (4.4%) specificities as unacceptable when the consensus was acceptable. Discrepancies occurred for specificities in all HLA loci with no clustering within a particular locus.

## 4 DISCUSSION

The aim of the EUROSTAM project is to analyse the feasibility of starting a Europe‐wide acceptable mismatch programme in order to enhance transplantation of HSPs with rare HLA phenotypes in relation to their own donor population. A prerequisite for a fair prioritized allocation of donor kidneys to these patients via an acceptable mismatch programme is a uniform definition of acceptable mismatches. The current policy within Eurotransplant is that eligibility of the patients for the acceptable mismatch programme and definition of acceptable mismatches are validated by the Eurotransplant Reference laboratory.

Findings in the present study confirm previous publications on the inter‐laboratory variability of Luminex assays^11^, ^12^, ^13^ although the study has still several limitations as basis for a successful Europe‐wide acceptable mismatch programme. The analyses were solely based on antibody testing results; AM designations were not performed in the context of transplant recipients. Repeat mismatches of previous transplants, and partner mismatches in the case of female patients, were not taken into account. The role of HLA‐DQA and –DP‐specific antibodies has not been considered in this pilot study, although these are certainly relevant for the allocation of donor kidneys to HSPs. The same holds true for allele‐specific antibodies, which may be a reason to consider high‐resolution typing of patients and donors in a future Europe‐wide acceptable mismatch programme,^14^ especially as the frequency of the alleles may differ among the different European populations.^7^

The observed variation in the definition of acceptable mismatches is not only due to differences in laboratory procedures or technical aspects of the assay. It is clear that the interpretation of the same results still leads to discrepancies. As expected, the degree of agreement was the highest in cases where the MFI values were very low or very high, whereas most discrepancies were observed in cases where the MFI value was between 1000 and 2000. All the laboratories have their own protocols and policies tailored for their particular clinical team. The interpretation of a given set of results and the appetite for risk are influenced by the environment created by the clinical protocols, a universal protocol and a strict MFI cut‐off value for assigning specificities in general is not practical. However, in the case of participation in a Europe‐wide acceptable mismatch programme, a high level of consensus is crucial both for the definition of eligible patients and the allocation of kidneys on the basis of acceptable mismatches. Although centres can decide whether to accept or decline organs based on their own protocols and appetite for risk, a final check by a central facility, as is currently the case for the Eurotransplant acceptable mismatch programme, will increase fairness and transparency of a future Europe‐wide acceptable mismatch programme.

## DISCLOSURE OF INTERESTS

The authors have declared no conflicting interests.

## AUTHOR CONTRIBUTIONS

M.C., Y.Z., D.R., J.M., D.M., A.S., A.I., F.C., S.F. designed and performed the study. Y.Z., D.R., F.C. contributed important reagents. M.C., Y.Z. collected data for the study. M.C., F.C., S.F. analysed the data and wrote the paper.

## Data Availability

The data that support the findings of this study are available upon reasonable request from the corresponding author, M.C.
